# Can narrative help people engage with and understand information without being persuasive? An empirical study

**DOI:** 10.1098/rsos.231708

**Published:** 2024-07-10

**Authors:** Alexandra L. J. Freeman, Lisa-Maria Tanase, Claudia R. Schneider, John Kerr

**Affiliations:** ^1^ Winton Centre for Risk & Evidence Communication, DPMMS, University of Cambridge, Wilberforce Road, Cambridge CB3 0WA, UK; ^2^ Department of Psychology, University of Cambridge, Downing Street, Cambridge CB2 3EB, UK; ^3^ School of Psychology, Speech and Hearing, University of Canterbury, Private Bag 4800, Christchurch 8140, New Zealand; ^4^ Department of Public Health, University of Otago, Wellington, 29 Brandon Street, Wellington 6011, New Zealand

**Keywords:** narrative, persuasive, communication, evidence communication

## Abstract

Stories have been shown to be engaging and aid the comprehension and retention of information. However, the persuasive power of storytelling is well-recognized. Is this an inherent property? Can a narrative be constructed that helps people engage with information but does not persuade them? We presented participants (*n *= 1309) with information about a fictional new drug and asked them whether they would license it on the basis of this. All saw the same information, in either a bullet-pointed list or as a ‘process narrative’—a journalist’s ‘journey of discovery’, designed to avoid persuasive language. Participants rated the narrative format a little more engaging than the non-narrative (*p* = 0.033, *d* = 0.12) and remembered the information in it slightly better (*p* = 0.040, *d* = 0.11). They did not rate the narrative version as more persuasive, but those reading it were on average more opposed to licensing the drug than those reading the non-narrative (*p* < 0.001, *d* = 0.18). Based on participants’ responses to other questions, we speculate this may be owing to the increased salience of risks of the drug, arising from subtle differences in wording. Thus, while narratives may have useful properties, they must be carefully constructed to avoid unintentional effects.

## 1. Introduction

Narratives play a central role in human communication and do more than communicate information. They characteristically have a structure that relies on temporal elements (things happen at specified or implied times relative to each other) or causal elements (one thing leads to another) and a personal voice (either as a character within the action or a narrator outside it) rather than simply being a collection of pieces of information, such as a bullet-pointed list. Stories can convey information and meaning, and they can also potentially change their audiences’ attitudes or behaviours—and it is possible that, because of their structure and the effects this has on the way we process them, they do this in a way that is different from non-narrative forms of communication.

The use of narratives in the professional communication of factual information is common. For example, presenting a particular patient’s story is often seen as a way of helping people understand information that they need in order to make a medical treatment decision [1]. This kind of information provision is designed to support the patient in making an autonomous decision, informed by the factual information and influenced by their own values and priorities [2]. However, most research into the psychological properties of narratives has been concentrated in the field of ‘narrative persuasion’—using the emotional engagement that narratives can create to change the audiences’ minds [3,4]. There is much less research into the question of whether it is possible to construct an engaging narrative that improves comprehension *without* persuading, in circumstances where it is important that the information format does not end up swaying the audience’s perceptions of the issues towards one particular outcome, as would be important in, for example, a patient decision aid. This kind of communication is called evidence communication, where the aim is to provide the audience with information to assist them in making their own decisions, for moral, ethical or legal reasons (e.g. ensuring informed consent in medicine or describing forensic evidence in court) [5].

The reason narratives are used in these contexts is to help readers connect with the factual information and imagine potential future scenarios [6]. A recent review of empirical evidence for these outcomes by Shaffer *et al.* [7] found that narratives did indeed increase comprehension and reduce misunderstandings when compared with an equivalent piece of non-narrative factual text. For example, a study aiming to improve recall of opioid-prescribing guidelines by Kilaru *et al*. [8] showed that not only was recall significantly greater in the narrative arm than in the non-narrative arm, but there were significantly more falsely recalled pieces of information in the non-narrative arm than the narrative arm. There are aspects of narrative structure that might help explain these sorts of findings. Faster reading times for narratives suggest that narrative sentences are easier to comprehend and integrate, possibly because integration is aided by the temporal and causal connections [9–11], as well as readers having a greater familiarity with, and seeing predictability in, narrative structure [12]. Personalization (as defined by Glaser *et al*. [9]), may also aid comprehension by making information easier to process and more relevant and contextual because narratives emulate everyday experience and are, therefore, easier to put into a human perspective [13].

However, narrative may be a double-edged sword. Indeed, narratives have been used intentionally to sway attitudes on a range of scientific issues, such as vaccines [14], or HIV/AIDS [15]. This is overt ‘narrative persuasion’, but persuasion can also be unintentional. While early works on persuasive communication defined persuasion as any message that is intended to shape, reinforce or change the responses of others [16], more recent efforts [3,17–20] have shifted focus away from intent to the process and outcome (e.g. what factors can make the same content more or less persuasive).

Reviews of the use of personal stories in patient decision aids have highlighted the complexity of the different types of narrative and the different effects on audiences recorded in a variety of different audiences and circumstances [21,22]. The type, structure and content of personal stories may either bias decisions and persuade, or facilitate objective informed decision-making: the design and style of narrative can make the difference between a narrative that informs, and one that also persuades—even unintentionally.

For those who wish to inform their audience without persuading them to align their beliefs with that of the protagonist or the communicator, then, are there ways to create a non-persuasive narrative with only the benefits of engagement and transmission of factual material? In the broader landscape of evidence communication [5], as stressed by Dahlstrom & Ho [13], these considerations intersect with beliefs on the appropriate roles of evidence communication within democracy. Being able to improve the communication of factual information around personal or societal issues through the use of narrative formats without inevitably invoking persuasion could be an extremely useful addition to the evidence communication toolbox. If, however, it turns out not to be possible to use narrative in a way that is non-persuasive, then this should also raise caution to those who work in areas where persuasion is not permitted or discouraged, such as medical communication, expert communication around evidence in court, or within the civil service.

Narrative persuasion can happen through different means, and even a narrative aiming for objective and neutral content and format and designed solely to improve comprehension may still sway the audience’s views in one specific direction without intending to do so. In the review by Shaffer *et al.* [7], the majority of reviewed studies were focused on persuasion, i.e. changing the audiences’ beliefs and behaviour, but not all of this was anticipated. For instance, a study by Betsch *et al.* [23] showed that narratives describing negative side effects associated with vaccinations increased the vaccination risk perception and reduced intentions to vaccinate, which was unintentional. Generally, in fact, narrative information was found to have a greater effect on the perceived risk and behavioural intentions than the statistical risk information in the decision aids, suggesting a higher relative persuasiveness of narratives as a format.

However, another way that narratives could persuade is through a simple imbalance in content. For example, one of the reviewed decision aids [24], despite aiming to respect the impartiality norms of the International Patient Decision Aids Standards, did not make it possible to compare the positive and negative features of the available options and focused on the positive experience of screening, while omitting any negativity or discomfort, creating an imbalance in the content. In fact, another review found that out of 30 personal stories in publicly available patient decision aids, 70% contained only stories portraying satisfaction, probably influencing the reader by focusing on the potential benefit and omitting the potential drawbacks unwittingly [1].

Balance, of course, does not mean false balance: decision aids regulators have expressed concern that even a ‘balanced’ presentation of views in a narrative can potentially give false impressions that there is an equal split in opinion about treatment, when in fact a large majority of patients might recommend or accept a particular option [25]. In a study by Ubel *et al*. [26], participants were presented with hypothetical statistical information about the percentage of angina patients who benefit from bypass surgery (75%) and given written testimonials from hypothetical patients who had benefited or not benefited from the treatment. The numbers of patients benefiting and not benefiting were varied to be either proportionate to the statistical information or disproportionate. The study assessed their choices of hypothetical treatment options when exposed to a fifty-fifty share narrative (participants receiving this disproportionate questionnaire version received only one testimonial from a patient who benefited from surgery and one from a patient who did not) or exposed to a narrative proportionate to the statistical information (participants receiving the proportionate questionnaire version were given three testimonials from patients who benefited from bypass surgery and one from a patient who did not, consistent with the hypothetical statistical information). Surgery, perhaps not surprisingly, was preferred more often by participants receiving the proportionate version than by those receiving the disproportionate (‘false balance’) version.

Even if narratives are balanced in content (proportionately, not ‘false balance’), there might still be persuasive effects—stemming from properties inherent to narratives. There are clues in the literature as to how some narratives might be designed in a way that makes them more persuasive than a non-narrative presentation of the same information, and hence what might need to be avoided in a ‘non-persuasive’ narrative form.

Theories of narrative persuasion ascribe the persuasive effect of narrative formats to specific mechanisms through which narratives are believed to inhibit informed decision-making. For example, research suggests that information processing of narratives tends to be less analytic and triggers more affective mechanisms rather than cognitive processes, relying on the employment of heuristic rather than systematic screening strategies when compared with didactic information [27,28]. The Elaboration Likelihood Model (ELM) of persuasion [29] recognizes that the degree to which readers ‘elaborate’ mentally on the arguments and evidence presented to them affects the degree to which they are persuaded by it, and there are many ways in which their degree of elaboration and then ultimate decision-making can be affected (e.g. [30]). When readers do not elaborate on the information and rely on heuristics, this may lead to lowered scrutiny and questioning of the information. Indeed, empirical studies report that audiences exposed to narratives produce fewer counterarguments than those exposed to other forms of communication [28,31]. It is possible that the internal coherence defining a narrative (such as the implications of causality between events within it) in some way inhibits the reader from employing full critical attention [32]. As such, narratives may sway people in one direction because their content is not offering explicit advocated positions, but simply showing a course of actions happening to characters facing specific problems. These narrative contents focusing on one specific case and lived experience make no assertion about how representative or typical the case is but show vividly specific people in specific situations, times and spaces, transporting the reader [33]. People may engage with this set viewpoint, or align their opinions with those of a character they identify with [34], without critically thinking about them or questioning their validity.

Turning to the empirical literature, lack of consistency in definitions of narratives, and stylistic and content differences between empirical studies, make it difficult to draw conclusions about the effects of ‘narrative’ compared with the effects of other elements of the communications being tested, such as the level of emotionality in language, balance of content, proportions of mentions of benefits and risks for either treatment option, presence or not of framing effects and other content or stylistic variations.

For the purposes of clearly defining narrative in the current study, we draw on several previous definitions. Dramatization (causality and temporality) and personalization (character/protagonist) are generally believed to distinguish narratives from other forms of communication [9,35–38]. The review provided by Hinyard & Kreuter [38] defines narrative as ‘any cohesive and coherent story with an identifiable beginning, middle, and end that provides information about scene, characters, and conflict; raises unanswered questions or unresolved conflict; and provides resolution’. Dahlstrom [39] refers to narratives as following ‘a particular structure that describes the cause-and-effect relationships between events that take place over a particular time period that impact particular characters’. Glaser *et al*. [9], describe the key defining features of narrative as being ‘personalization’ (including an individual voice or character to communicate the salient facts) and ‘dramatization’ (a structured approach to giving factual information, such as a timeline or sequence that describes cause and effect). We, therefore, use a definition of narrative that includes three features: causality, temporality and character.

Empirical work suggests that the causal and temporal structure of narratives—a ‘one thing leads to another thing’ approach (referred to as ‘dramatization’ by Glaser *et al*. [9]) could have intrinsic comprehension benefits in the context of evidence communication. For example, studies in science communication suggest that audiences find dramatized narrative structures more engaging and easier to understand than what might be called traditional, logical-scientific communication [40–42]. As outlined above, personalization is also theorized to lead to comprehension benefits [13].

There is also evidence that a particular types of narrative structure using these dramatization and personalization elements may serve our purposes better than others. Shaffer & Zikmund-Fisher [43] carried out a systematic evaluation of narratives used in medical decision aids, providing a framework defining different narrative types. They define three dimensions that they consider likely to moderate a narrative’s impact on decision-making: (i) the purpose of the narrative, (ii) the content of the message, and (iii) the evaluative valence, or overall tone, of the message. Other authors have emphasized the importance of the information content of the narrative on its effect on the audience [7,22] and de Graaf *et al*. [44] found that different types of emotion-laden narratives manipulating character identification had different effects on behaviour in their study. Shaffer *et al*. [45] suggest as a result of their review that what they call ‘process narratives’—stories that focused on how someone made a particular decision rather than their actual final decision—increased the time the audience spent searching for information in a patient decision aid for early stage breast cancer, indicating higher engagement and more focus on the information. Such a narrative design appears a promising option for science communicators seeking to inform without persuading and so is the one chosen for this study.

There are divergent views about whether additional elements should be considered as defining features of narratives or stylistic elements of particular types of narratives, and these divergences could contribute to the potential persuasive effects of narratives. For example, there is a disagreement on whether emotional language is an integral part of narratives by definition or merely an additional stylistic choice. Olson [46] believes that narratives are ‘facts wrapped in emotions’, and hence, the emotional content of the language may be an inherent and important element of narrative, making them inappropriate for those seeking unbiased communication. Studies in the field of persuasive narratives, indeed, sometimes use the terms narratives and emotional stories interchangeably by distinguishing the narrative arm from the non-narrative arm through the presence of emotional language evoking the worry, fear or sadness of the protagonist in the narrative treatment [47,48]. However, emotionality of language has repeatedly been associated with increased persuasiveness or as a mediating factor of narratives’ persuasiveness, and so we attempt to avoid overuse of emotion in our study materials while hoping that this does not undermine the engagement properties of narrative that we hope to retain.

Another consideration, as already mentioned, is the degree of balance in the information content of the narrative. Many narratives presented to participants in studies on narrative persuasion are written with a clear imbalance of content (e.g. content only focusing on the benefits of a treatment, but no content on the risks of the treatment). These studies most of the time do not acknowledge the potential causal role of the unbalanced content and fully ascribe the persuasive effect to the narrative format. Most narratives in the literature have been operationalized as being one-sided in content [1], leading to the question of whether a narrative presenting information in a balanced way without including content pointing at a clear ‘best’ option, but instead presenting both sides, would still lead to the same persuasive effect. Assessing whether a narrative presenting balanced information could be non-persuasive and, therefore, be appropriate for those seeking impartial science communication constitutes a gap in the literature.

If the majority of narratives currently used in studies form an accurate representation of what defines narratives, it seems that both their emotionality and one-sided content are likely to lead to persuasion, but it is not clear whether designing a narrative without these features could avoid persuasion.

Building on the existing empirical evidence and theory, then, a ‘process narrative’, with carefully balanced information and minimal emotionality, seems the type most likely to help us answer our key question: is it possible to construct a narrative (with personalization and dramatization) that demonstrates the engagement and comprehension benefits of the format (over a non-narrative comparator) but does not persuade readers towards one conclusion or another? In the current study, we attempt to design a ‘process narrative’ (and for comparison, a non-narrative) presentation of the same information to identify whether or not it is possible to construct a non-persuasive narrative that retains the advantages of the narrative format identified in previous studies: increased engagement and comprehension. This would help guide future evidence communicators identify ways of presenting factual content for their audiences when aiming to inform but not persuade.

## 2. Hypotheses

**Hypothesis 1 (H1):** We hypothesize that a narrative version of a non-narrative (bullet-pointed) set of facts can be constructed that (a) does not lead the audience to one particular decision over another (i.e. is non-persuasive) and (b) is not perceived as more persuasive towards a particular choice, compared with a non-narrative version.
**Hypothesis 2 (H2):** We hypothesize that such a narrative version of the information would result in both (a) higher recall and (b) higher comprehension of the facts than the equivalent non-narrative version.
**Hypothesis 3 (H3):** We hypothesize that such a narrative version of the information would be rated as more engaging by the audience than the equivalent non-narrative version.

## 3. Methods

In order to test the effects of a narrative versus a non-narrative format, we carried out a randomized two-arm, between-subjects experimental study, presented online using the survey builder Qualtrics.

We created two short written materials, situated in a medical context (the hypothetical licensing decision around a new drug), both designed to be non-persuasive with no strong emotional language and with balanced information, i.e. approximately balanced potential benefits and risks. See electronic supplementary material, appendix A for final versions, and the repository on OSF for previous versions used in two pilot studies to help us refine the material and methods (https://osf.io/9jsvy/).

The ‘narrative’ version of the information is a ‘process narrative’ (‘journey of discovery’) (dramatization) with the author and their friend (a sufferer of the disease in question) as protagonists (personalization). The non-narrative format presents the information in a series of bullet points designed to be clear but lack the defining features of a narrative.

Information was kept constant across the two arms, ensuring that both materials contain the same factual information, to avoid confounds between the experimental groups (table 1). The lengths and readability of the different materials were kept approximately equal (Flesch-Kincaid Grade level 10.2 (narrative) and 10.38 (non-narrative), reading age 15–16 [49]). For the narrative condition, strongly emotional language or opinions of the protagonists were avoided. However, we featured both personalization (a protagonist narrating their experience of looking at benefits and risks, on behalf of a named friend who suffers from the disease) and dramatization (a causal and chronological flow from one goal or question about the treatment to the next), in accordance with the definitions by Glaser *et al*. [9].

**Table 1.** Experimental conditions.

	narrative condition	non-narrative condition
elements common to both conditions	– scenario of a fictional new drug being presented for a decision on licensing by the relevant health authority – symptoms described of a fictional disease (Ziegler disease) in the same terms – pros and cons of a fictional new drug (BenoZenad) described, including the outcomes of clinical trials, giving the same framing and emotional salience of the information	
elements differing between conditions	format: narrative text as a ‘journey of discovery’ or ‘process narrative’ (dramatization) with the author and their friend as protagonists (personalization)	format: bullet-pointed text laid out to resemble the format of communication of the European Medical Agency summary reports

Participants were randomized, using the ‘randomize’ function of Qualtrics, to be presented with one of the two materials. After the initial presentation of the materials, they were asked a series of questions, ending with demographic questions about themselves. The amount of time they took to read the information initially, and to carry out the objective comprehension questions, was recorded.

### 3.1. Measures

Our main dependent variables are the degrees to which the materials persuade audiences in their beliefs, recall, comprehension and engagement.

#### 3.1.1. Persuasion

To investigate whether the information persuaded participants, we asked them to make a decision based on the information immediately after they read it (‘Based on the information you have just read, would you license BenoZenad if you were part of the decision-making committee?’—BenoZenad being the fictional new treatment described in the information). Participants were given a 0–100 slider with the anchors ‘disapprove strongly’ (0) and ‘approve strongly’ (100) with ‘can’t decide’ as the midpoint (50). They were also asked to give their reasoning in free text.

We used this measure to test H1a, hypothesizing that there would be no difference between conditions.

Later in the survey, participants were asked a set of questions to assess whether they perceived the information to have persuasive intent (‘Do you think the message aimed to persuade or inform you?’ (anchored ‘persuade’ (0) and inform (100)) and ‘Was the information one-sided or balanced?’ (anchored ‘one-sided (0) and balanced (100))—both answered by sliders; ‘To what extent do you feel that the information you just read was aiming to persuade you?’ and ‘To what extent did the text provide a fair account of BenoZenad’s harms and benefits (as opposed to concentrating more on benefits or more on harms)’—both answered by seven-point Likert scales (strongly disagree (1) to strongly agree (7)); and the two-question persuasion perception scale [50]: ‘The person who wrote the text wants to convince me of a particular point of view’ and ‘The person who wrote the text wants to influence their reader’s behaviours’, both again answered by seven-point Likert scales. The Likert scale items were rescaled (0–100) and reverse coded as appropriate, then a mean of the responses to all six questions was used to create an index of perceived persuasive intent.

We used this index to test H1b, hypothesizing that there would be no difference between conditions.

#### 3.1.2. Recall

Immediately after the first questions about the decision they would make after reading the information, participants were tested on their recall of facts through 10 randomized multi-choice questions based on facts (both gist and exact recall) given in the texts. Participants were given a score out of 10 representing their recall.

We used this score to test H2a, hypothesizing that it would be higher in the narrative condition.

#### 3.1.3. Comprehension

We used an objective test of comprehension in which participants were shown the information again and a series of five factual multi-choice questions directly underneath it. Participants were given a score out of 5 representing their comprehension.

To test H2b, we compared objective comprehension scores between conditions, hypothesizing that they would be higher in the narrative condition.

#### 3.1.4. Engagement

Engagement in the information was measured with a set of three questions (‘In your opinion, would other people want to read this?’, ‘Were you interested in the text?’ and ‘Were you bored while reading the text?’—each answered with a seven-point Likert scale). The mean of all three items was taken to form an index of engagement.

We used this index to test H3, hypothesizing that engagement would be higher in the narrative condition.

Secondary measures included: perceived trustworthiness, a subjective measure of the comprehensibility of the information presented to participants [51], the immersion scale adapted from Wojcieszak & Kim [52] and a series of questions to assess aspects of the balance of the information presented (in terms of factual content, technical language and emotion). See electronic supplementary material, appendix B for a full list of variables.

We included one attention check (‘So that we know that you’re concentrating, please select ‘very much’). Participants who failed the attention check were excluded from the analysis.

These questions were all piloted in a pre-registered pilot study of 161 participants in order to assess any potential ceiling effects. After minor adjustments were made to the two stimuli, a second pilot study of 162 participants was run (see https://osf.io/9jsvy/ and electronic supplementary material, appendix C).

### 3.2. Statistical analyses and power

Using GPower to estimate the necessary sample size for a two-sample, two-tailed *t*‐test on our primary dependent variables, with a small effect size of *d* = 0.18 gives *n* = 1300 for 90% power.

All dependent variables were ‘forced response’ in the questionnaire, avoiding missing data.

All analyses were conducted in R.

### 3.3. Participants

We used the participant recruitment company Bilendi & Respondi (https://www.bilendi.co.uk) to recruit participants to the online survey. Participants were paid the company’s standard rate for completing a 15 min survey. We set quotas to ensure that the participant pool was broadly representative of the UK population as a whole on age and sex according to the most recent national census. Quotas were not decremented for participants who failed the basic attention check (*please select ‘very much’*), and data from these participants were excluded from analysis, as pre-registered.

A total of 1680 participants completed the survey, and 371 (22.08%) failed the attention check. The attention check failure rate did not differ significantly between conditions (*X* ^2^(1) = 2.57, *p* = 0.11). This resulted in a final analytic sample of 1309 attentive participants. Participants were randomized to read either the narrative (*n* = 692) or non-narrative (*n* = 617) version of the text. The average reading time in the narrative condition was 3.63 min (s.d. = 5.20) and 2.31 min in the non-narrative condition (s.d. = 2.56). Sample demographics are given in table 2.

**Table 2.** Sample demographics.

category	response	*n*	(%)
sex	female	667	51
	male	637	48.7
	prefer not to say	5	0.4
age	18–24	157	12.0
	25–34	251	19.2
	35–44	235	18.0
	45–54	247	18.9
	55–64	232	17.7
	65+	187	14.3
education	no formal education above age 16	143	10.9
	professional or technical qualifications above age 16	195	14.9
	school education up to age 18	319	24.4
	degree (Bachelors) or equivalent	425	32.5
	degree (Masters) or other postgraduate qualification	211	16.1
	doctorate	16	1.2

We pre-registered our hypotheses and analysis plan (https://doi.org/10.17605/OSF.IO/GZS8Y). H1a was pre-registered to be tested by using participants’ decision on licensing. H1b used the index on perceptions of persuasion created from participants’ answers to six questions if the items correlated sufficiently (Cronbach’s α > 0.6; in the event this threshold was not met we pre-registered that we would remove up to two items, based on the lowest item-total correlation, to improve reliability to α > 0.6). In each case, we pre-registered that we would first use a *t*‐test, and if there was no significant difference between condition means, follow up with two one-sided tests (TOST) equivalence test with α = 0.05, assuming equal variances, and equivalence bounds of *d* = −0.1 and *d* = 0.1 [53].

H2 was pre-registered to be tested by comparing participants’ mean recall scores (H2a) and comprehension scores (H2b) between conditions using Welch’s *t*‐test [54].

H3 was pre-registered to use the index on engagement created from participants’ answers to three questions if the items correlate sufficiently (Cronbach’s α > 0.6) to test for difference in means between conditions using Welch’s *t*‐test [54].

The Stage 1 Registered Report accepted manuscript can be viewed at https://doi.org/10.17605/OSF.IO/9JSVY.

## 4. Results

Hypothesis 1 posited that the narrative and non-narrative texts would not differ in terms of persuasion. We set out to test persuasiveness in two ways: objective persuasion in terms of the decision participants would make as a result of reading the material (H1a) and subjective perception of persuasion (H1b).

Participants in the non-narrative condition expressed greater support for licensing BenoZenad (*M* = 63.13, s.d. = 24.93), compared with those in the narrative condition (*M* = 58.41, s.d. = 26.74; *p *< 0.001, *d* = 0.18). Therefore, H1a was not supported.

In terms of the perceived persuasive intent of the messages, we used an index measure as described (α = 0.78).

**Figure 1.**
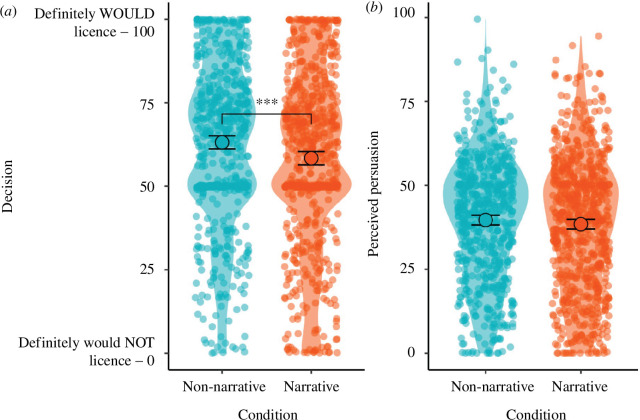
Participant support for licensing BenoZenad (*a*) and perceived persuasive intent of the message (*b*). Solid points and error bars represent the mean and 95% CI. Violin plots and jittered points display the distribution of underlying data. Brackets denote a significant difference between conditions, *** *p *< 0.001.

There was no significant difference between the non-narrative (*M* = 39.68, s.d. = 18.33) and narrative (*M* = 38.46, s.d. = 19.57; *p* = 0.24) conditions on the perceived persuasion index, where a higher number indicates a higher perception of persuasive intent. However, as pre-registered we conducted a follow-up equivalence test using the two one-sided tests (TOST) procedure, based on the smallest effect size of interest of *d =* 0.1 (in either direction). We obtained a non-significant result of *t*(1303.86) = −0.64, *p* = 0.26, for the lower bound. Therefore, while the means did not differ significantly between conditions, we cannot conclude that they are statistically equivalent. See figure 1 for the data on hypothesis 1.

Hypothesis 2 posited that participants reading the narrative text (versus non-narrative) would perform better on tests of recall (H2a) and comprehension (H2b) of information.

Participants in the narrative condition scored significantly higher on a test of recall (*M* = 5.80, s.d. = 2.22) compared with participants in the non-narrative condition (*M* = 5.55, s.d. = 2.23; *p* = 0.040, *d* = 0.11), supporting H2a.

**Figure 2.**
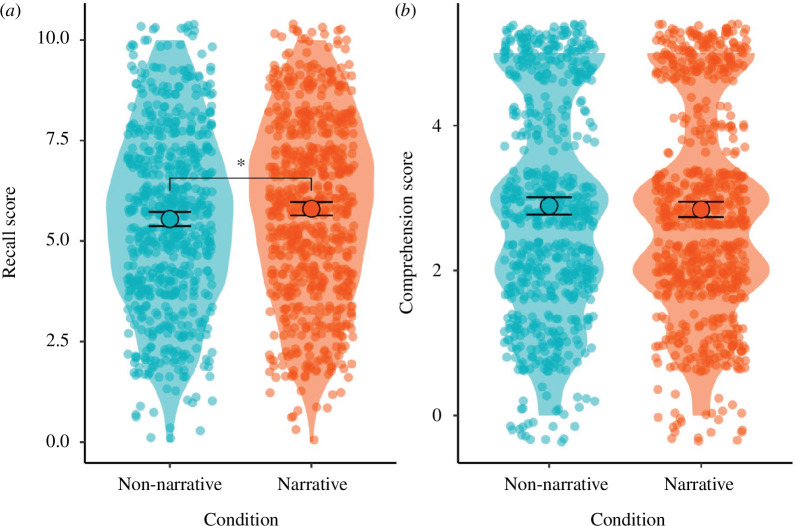
Participant scores on recall (*a*) and comprehension items (*b*). Solid points and error bars represent the mean and 95% CI. Violin plots and jittered points display the distribution of underlying data. Brackets denote a significant difference between conditions, * *p *< 0.05.

Considering comprehension scores (where participants had access to the text while answering questions) there was no significant difference between the narrative (*M* = 3.06, s.d. = 1.32) and non-narrative (*M* = 3.01, s.d. = 1.38; *p* = 0.482) conditions. Therefore, we did not find support for H2b. See figure 2 for results relating to hypothesis 2 and electronic supplementary material, appendix D for participants’ performance on each individual question.

Hypothesis 3 posited that the narrative text would be rated as more engaging than the non-narrative text.

**Figure 3.**
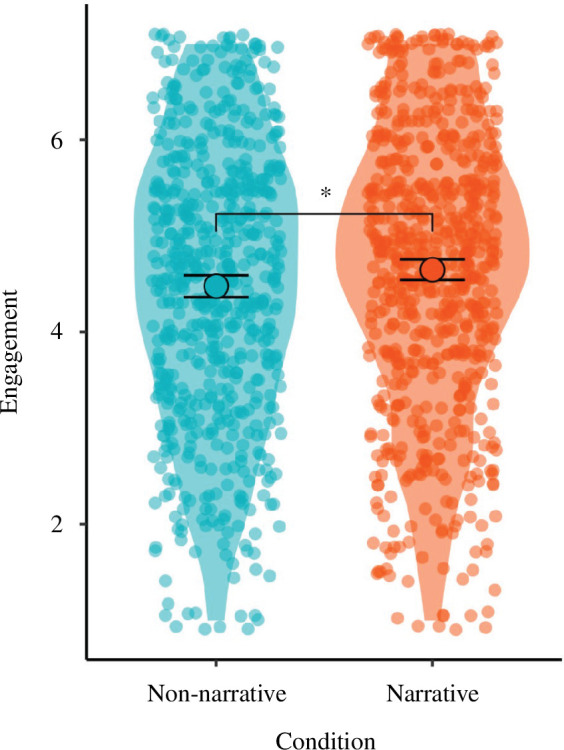
Ratings of engagement with text. Solid points and error bars represent the mean and 95% CI. Violin plots and jittered points display the distribution of underlying data. Brackets denote a significant difference between conditions, ** *p *< 0.01.

We used an index measure of engagement (*α* = 0.82). Participants in the narrative condition rated the text as more engaging (*M* = 4.65, s.d. = 1.43) than those in the non-narrative condition (*M* = 4.48, s.d. = 1.45; *p* = 0.033, *d* = 0.12) on this index, supporting H3. See figure 3.

A summary of the results of both primary and secondary measures is listed in table 3. See electronic supplementary material, appendix D for graphs of the data on each.

**Table 3.** Outcomes for all measures.

outcome	α	non-narrative		narrative		*p*	*d*
		*M*	s.d.	*M*	s.d.		
decision		63.13	24.93	58.41	26.74	**<0**.**001**	**0.18**
perceived persuasion (index)	0.78	39.68	18.33	38.46	19.57	0.244	0.06
recall score		5.55	2.23	5.80	2.22	**0.040**	**0.11**
comprehension score		3.01	1.38	3.06	1.32	0.482	−0.04
engagement (index)	0.82	4.48	1.45	4.65	1.43	**0.033**	**0.12**
immersion (index)	0.81	4.17	1.25	4.49	1.28	**<0.001**	**0.26**
trustworthiness (index)	0.92	5.20	1.22	5.08	1.23	0.077	0.10
perceived benefits of BenoZenad versus AvalonZ		65.81	21.59	66.08	21.8	0.825	0.01
perceived risks of BenoZenad versus AvalonZ		58.79	23.2	63.68	23.07	**<0**.**001**	**0.21**
subjective understanding		4.84	1.51	5.08	1.48	**0.004**	**0.16**
effort required		4.95	1.54	4.88	1.58	0.389	0.05
difficulty		3.58	1.67	3.44	1.7	0.131	0.08

Note: Significant (*p* < 0.05) effects bolded

As listed in table 3, participants in the narrative (versus non-narrative) condition, on average, scored higher on our measure of immersion (see electronic supplementary material, appendix B). They also perceived greater risks of BenoZenad and had higher self-ratings of understanding the text.

Participants in the narrative condition also spent longer, on average, reading the material provided. See electronic supplementary material, appendix D for additional, non-pre-registered, analyses of the effects of reading time on outcomes which were suggested during the review process.

## 5. Discussion

We set out to investigate whether it was possible to create a narrative format that engaged people, helped them understand and remember information, but did not persuade them one way or the other when making their own decision based on that information.

The narrative format was not deemed by participants to be subjectively more persuasive than our control, a series of bullet points (although the TOST test could not confirm that the two were not significantly different in terms of perceived persuasion, it was the non-narrative format that had the slightly higher mean persuasiveness rating). However, those who read the narrative version on average reported that they would be less likely to license the new fictional drug BenoZenad. Responses to other questions, and the free text responses on why they made their decision, give us a possible sense of why that is. Those reading the narrative format rated the potential benefits of BenoZenad the same as those reading the non-narrative version, but they rated the potential risks higher. The relevant sentences from the two formats are: ‘the trial did also record a significant difference in the risk of blindness (2% in the BenoZenad group, 0.5% in the AvalonZ group)’ (non-narrative condition) versus ‘the most common serious side effects… also included blindness (2% in the BenoZenad group, 0.5% in the AvalonZ group)’. The difference in the phrasing here may be the cause of the difference in perception of the risks, as one participant said: “significant difference in the risk of blindness’ I am unsure what this meant. Did the drug cause some people to go blind?” When we asked, in the recall questions, which drug caused more cases of blindness, 34.4% of those in the narrative condition selected the answer that ‘blindness was not reported as an adverse event from the drugs’, as opposed to 27.5% in the non-narrative condition (see electronic supplementary material, appendix D).

When we were designing the studies and preparing the materials, we had an in-depth discussion about the difference between ‘persuasion because of the facts’ versus ‘persuasion because of the presentation format of the facts’. Although we had tried to keep ‘the facts’ and their salience the same, it appears from these results that the oversight of using the phrase ‘increased risk of blindness’ (as is common in medical results descriptions) in the non-narrative format might have meant that some people reading it did not realize that this meant that blindness was a potential side-effect of the drug and hence their perception of the risks of it was lower, and this then might have affected their decision over licensing. This is a salutary lesson to all of those trying to write informative information for a lay audience.

We must also consider the possibility that the narrative format made the risks more salient and ‘real’ to the reader. Emotional feelings may serve as a heuristic for a person’s overall assessment of situational risks and benefits [55], and participants did rate the narrative text as containing significantly more emotional language (see electronic supplementary material, appendix D, figure A4-3). Studies suggest that emotionalization of narratives can influence risk perception [56] and this difference in style— perhaps difficult to divorce from a narrative if one defining characteristic of narrative is personalization—could, therefore, have made the negative effect of blindness in this example more vivid.

Our other results, despite small effect sizes, indicate that a narrative format is indeed rated more engaging and easier to understand by participants, and scores higher on the ‘immersion’ index [52]. Importantly for communicators wanting to inform their audiences, the narrative format also scored higher on average on the recall questions. Results for each of the ten questions individually (electronic supplementary material, appendix D) confirm that this result was not caused by a single fact or two being considerably more salient in one format than the other (although it is interesting that the number of people with the fictional Ziegler disease was so much more memorable to those reading the non-narrative version). These findings support much of the previous literature on the properties of narratives [8,39].

Comprehension scores—based on questions asked while the text was available for participants to consult—showed no difference between the two formats. It is possible that participants found it onerous to go back through the text to find the answers, an explanation which is reflected in the relatively low scores on these questions, and some of the free text responses people left at the end of the survey.

Concerning the non-pre-registered exploratory additional analysis of reading time, it is impossible to draw conclusions about any effect in these findings owing to the design of this study. We did not manipulate reading time, rather, it is a measured variable as a function of the experimental condition. It is thus not possible to unpick the effects of reading time and condition. However, this could be an area for future studies to elucidate.

In conclusion, many writers will instinctively use storytelling to convey information to their audiences, and this study helps provide empirical evidence that this approach can ‘work’. It seems that it is possible to present factual information in a narrative style that can help people engage with and remember information, and which is not overtly persuasive in nature, and that a ‘process narrative’ (or ‘journey of discovery’) might be one way of doing that.

However, our results highlight just how difficult it is to avoid accidental changes in the salience of important facts simply through the use of unclear language, which can lead to people making different decisions based on their different understandings of the information presented, and the potential for increased emotionality, which might affect responses to the information.

For those who legally or ethically must not persuade their audience (such as those involved in medical informed consent) or for those who do not want to bring their audience’s emotions too strongly to the decision, this study shows the difficulty of using natural-sounding narrative language. Communicators wishing to inform and not persuade must always be very careful, whatever format and style of information they are using. When the readers’ responses are crucial, we would suggest running a survey using measures such as ours to test the effects before releasing the communication more widely.

## Data Availability

All data and analytical code relating to this study is available at [57]. Supplementary material is available online [58].
